# On the Mental Origin of Insanity

**Published:** 1830-09

**Authors:** Alexander Thomson


### INSANITY.

### *On the Mental Origin of Insanity.* By Alexander Thomson, M.B.

#### *The Primary and Universal Mental Cause.*

As we cannot compare more than two propositions at once, it follows that, unless the judgment upon these be accurate, (that is, be formed with a reference to all the possible relations of the two propositions,) any new judgment, resulting from a comparison in which the prior judgment forms one of the terms, will necessarily be *inaccurate.* Thus, supposing $x$ to be the judgment, which is the result of a comparison of $a$ with $b$, while the *accurate* judgment, or the conclusion to be arrived at, by all persons properly qualified for the examination, after a careful investigation, is $x'$: it is easily to be comprehended that X, the result derived from the $x$ compared with some other term $z$, will still partake of the original deviation from accuracy, however minute be the difference between $x$ and $x'$, the true or accurate result.

By a similar mode of reasoning, we may conceive how all propositions to be deduced directly from X, or from any proposition into the formation of which X has, either directly or indirectly, entered, will partake of the original or first error. But all our knowledge is founded upon sets of propositions compared by twos, so that every error, however small, in more comparisons than one, will combine to throw a cumulative cloud of error over the final conclusions which form, as we all know, the standards by which we measure the value of our acts; or, in common, the principles upon which we act, or the motives which govern us in our selection between every two modes of action presented for our choice or adoption.

Where such errors occur in only one or two of the comparisons furnishing the conclusions which form our knowledge, the deviations from the ordinary results obtained by others thence produced not being very great, the party is called *eccentric* or *peculiar*. When they are more numerous, the individual being bewildered between which of his conclusions may be accurate, and therefore depended on, and which may not be so, and being still induced to place implicit confidence on all his conclusions, reasons and acts apparently in so anomalous, abnormal, or untraceable a manner, that he obtains the epithet of *silly* or *foolish*. If, in addition to this folly, he be so conceited, so satisfied of having himself taken into consideration all the circumstances necessary to determine a strictly accurate result, that he will not condescend to reanalyze any conclusion at which he has arrived, however much that conclusion may differ from that arrived at by others, he is then justly styled *mad*.

That in this manner a man may reason inaccurately, that is, in a way in which no other man will, if capable of making and striving to be accurate in an investigation, from accurate premises, or from the same propositions as are compared by others, is pretty evident. Thus, supposing ($a$) and ($b$) are to be compared, and that, in order to arrive at the conclusion $x$, it will be necessary to consider five qualities of ($a$); if any circumstance prevent us considering more than four of these, our conclusion may be perfectly accurate for the qualities considered, but will certainly not correspond with the conclusion (judgment) of the person who has duly weighed the five qualities in question. If the mind of one individual were similar in all respects to that of others, it is probable that he would have instituted and followed up a similar series of comparisons, and therefore would have taken duly into consideration all the five qualities required to be examined. But variety of education, variety of temporary circumstances, whether these be mental or corporeal, may obscure, in the mind of the individual comparing, the necessity for the examination of the fifth circumstance, nay, even prevent him from being aware of the existence of the same, and yet he be as sane, as *compos mentis*, as any person in the world; and, as it appears that this may happen in the conducting of one series of comparisons, so it may evidently happen once or oftener in one or more of the series of comparisons, or, to use more ordinary language, in one or more of the chains of reasoning, or the processes of investigation, the conclusions or judgments derived from which form the principles of our knowledge or of our motives. Hence, even in minds which will readily be acknowledged to be sane, such omissions, unavoidable on the part of the comparer, may, as we have already seen, lead to deviations of principle, so widely deviating from the conclusions of other men, or of men in general, that they can be styled *eccentric*, or the *result of temporary insanity*. Here, then, we have *accurate reasoning* (in the ordinary acceptation of the term,) on *false premises*, that is, on premises not the same as those used by others in investigating the same question, though, as has been shown, perfectly accurate as far as regards the individual, who is, unfortunately for himself, placed under the influence of the modifying circumstances. But *accurate reasoning from false premises* has been given as one of the definitions, one indeed which has been pretty generally received as accurate, of *insanity*. It appears, however, that such a mode of reasoning may arise from circumstances totally independent of the mind in which it passes, even while that mind will be acknowledged to be perfectly sound. It follows, then, that this is not an accurate definition of insanity, that is, of a state of mind inconsistent with health or integrity, and even to those adopting it as accurate, that insanity may arise independently of the brain, although, indeed, disease of the percipient organs may be one of the modifying circumstances which induce the omission of certain considerations or links in a chain of reasoning.

That definition of insanity which makes it to consist in *false reasoning from accurate premises*, is equally untenable: for, if *a* and *b* are to be compared, and to obtain the result supposed correct, namely *x*, *a* and *b* may be the whole of the premises, yet, in reasoning or comparing, the whole of their relations may not be taken into account, or the whole value may not be given to any single relation in some one or more of the series of comparisons. But such an omission may equally arise from circumstances over which the mind of the comparer had no possible control, and indeed resolves itself into an omission similar in its origin to the one already considered. It would be, indeed, absurd to deny that imperfections in the nervous system may give rise both to accurate reasoning from false premises, and to false reasoning from accurate premises. But surely it is fair to deduce from the preceding investigation, that these errors of the mind may also take place independently of disease, either mental or corporeal.

It may be urged, however, that accumulations of erroneous conclusions, while they may lead in one or two instances to eccentric, or even inexplicable, acts, will not certainly long continue to do so; inasmuch as the commission of any such act, meeting with reprehension from those whom it may influence, will itself induce the perpetrator, on reflection, to reexamine the nature of the reasoning upon which the determination to perform it was founded. But to this I have to answer, that the majority of mankind do not analyse or reflect upon the mode of action of their own minds; that thence they are not aware of the great deviation, in principle or in conduct, that may arise from a very slight omission in reasoning, and consequently are habituated to place implicit reliance not only upon the accuracy of the impression conveyed by the senses, but also upon the necessary truth of the conclusions drawn from a comparison of these. Men, in general, therefore, not only treat with contempt any proposal to reinvestigate the origin of their principles, but also with indifference, as it would necessarily lead them into much laborious investigation, in a path to which they are by no means used, and to which they are on that account naturally disinclined. In nine cases out of ten, therefore, an erroneous act is referred to some unknown source, as to a want of consideration, to the result of passion, or to an impulse over which the individual has no control, to some error in the constitution of the individual's mind, to some sinful tendency produced by the deduction of the race from Adam, to the agency of some evil spirit, operating by a strong and irresistible temptation. It is most worthy of remark that, in all these ascribed origins of an erroneous act, there is one common feature, viz. that the origin of the act was out of the power, out of the influence of the consideration of the individual perpetrating the act. If, then, I propose to consider these acts as arising from conclusions stored in the form of principles, or leading-strings for action, in the mind, wrong, because formed without a due reference to all the circumstances from which they are deduced by other men, and yet formed, with every wish on the part of the examiner to arrive at truth, what more do I call upon these men to believe than is found in their own creed? All I ask is, that, in forming these principles, they shall grant me that they are right as far as the power of the individual to investigate goes, but wrong, because he is prevented by circumstances over which at the moment he has no control, from taking into consideration all the circumstances which might have altered and modified his conclusion.

It is true that we apply our origin of error at different points: they immediately preceding the committal of an act; I, in some anterior point of the series of comparisons from which the principle guiding his selection has been deduced. Yet, even in my way of regarding this origin, the very last comparison in the series may be the origin of the wrong conclusion. The actor must always, preceding his act, compare the value of two modes of conducting himself, either of remaining inactive or of proceeding to action: and, in his selection between these two modes, he must be guided by the agreement of one, or rather by his fancying an agreement of one of them with the object which he has mediately or immediately in view. This fancied agreement implies a standard or previous conclusion, with which the two alternatives or their tendencies may agree; and thence the error may be either in the formation of this standard, or in the omission of some considerations which might make the one agree more certainly than the other with such standard.

From such observations we see how easily an error may be overlooked, how great may be the influence even of a trifling omission on the ultimate tendency of our actions. When these errors have become numerous, when they have turned awry so many of the acts of an individual, that his friends are induced to suppose something wrong: they treat him with caution and reserve, limit their former confidence, recommend him means for improving his health, (though the person himself does not even suspect it to be destroyed,) endeavour to make him believe that he is labouring under false impressions, that he reasons falsely or too subtilely, in fine, that he is no longer fitted for society. The friendship which formerly stimulated him to exertion is gone; and the eye which met his with kindness, with confidence, and with love, is averted. He in vain turns to his own heart to look for the causes of the change, and he feels dreadfully humbled and oppressed by being left as an alien upon the world, to gain his pleasure from the resources of his own mind, to soothe his pain as best he may in solitude, and sunk beneath what he believes the galling finger of scorn. Is it surprising that, under such circumstances, the man turns upon himself for an investigation of his own acts and motives, and that, tracing no immediate, or to him recognizable, error in the conclusions at which he has previously arrived, trusts with confidence his reasoning powers and his senses, which he has never yet taught himself to doubt, that he still further concludes his friends to be capricious and unjust, and in his turn acts towards them with suspicion, distrust, and cunning, or with anger, malevolence, and revenge, according to the previous turn of his moral and intellectual education.

Is it to be wondered if, between this struggle of indifference to life and the necessity of living; if, in contrasting the difference between the present and the former state; if, in contemplating the supposed hollowness of human affection, the balance of the circulation, which was kept up by a judicious regularity of habits induced by a regard to the opinion of society and of friends, be destroyed, and disease, slow and lingering disease, unalleviated by hope, and stimulated by despair, take possession of the frame. The body may now, and does, become as irritable as the mind; the eye, which was fixed in deep thought, becomes glassed by blood, which is irregularly sent to the tired, worn out, and agitated sensorium; and the whole system or being is placed in circumstances which tend not only to perpetuate his former errors, but to prevent him still more from taking into consideration all the circumstances necessary to determine his acts. The blood, sent by the increased impulse of the stimulated heart, rushes, as in the man under the influence of wine or other great stimuli, through the brain, bringing rapidly and unconnectedly before him various past scenes and impressions, which so occupy and fill his mind as to prevent the remembrance of his usual principles, or standards of action, being brought under his consideration. While in this state, should some unfortunate circumstances accidentally occur to rouse and excite his attention, he acts upon the impulse of the moment, without appealing to the ordinary standards of his actions, which are obscured by his fever, and he performs acts which himself will be the foremost to condemn when he is calmed by the exhaustion consequent upon the agitation. The violence of his acts will be in proportion to the degree of his agitation.

It appears from what has been said, that insanity is by no means necessarily dependent upon disease of, or even upon irregularity in the circulation of, the brain itself, while there can be little or no doubt that either of these may be one of the circumstances which prevents the investigating power from being used to their utmost extent. Indeed, notwithstanding the zeal and ardour with which some respectable men have sought for indications of disease in the brains of persons considered insane, in many instances they have found none; and, where they have been fortunate enough to find them, they have not in any two cases been the same; and it is most undoubtedly true that every indication yet found has been met with in the brains of those who have died without ever having been considered insane, without ever having given, at least, any demonstrations of insanity.

---

